# Single-emissive-layer all-perovskite white light-emitting diodes employing segregated mixed halide perovskite crystals†

**DOI:** 10.1039/d0sc04508j

**Published:** 2020-09-25

**Authors:** Hongling Yu, Heyong Wang, Galia Pozina, Chunyang Yin, Xiao-Ke Liu, Feng Gao

**Affiliations:** Department of Physics, Chemistry and Biology (IFM), Linköping University Linköping 58183 Sweden xiaoke.liu@liu.se feng.gao@liu.se

## Abstract

Metal halide perovskites have demonstrated impressive properties for achieving efficient monochromatic light-emitting diodes. However, the development of white perovskite light-emitting diodes (PeLEDs) remains a big challenge. Here, we demonstrate a single-emissive-layer all-perovskite white PeLED using a mixed halide perovskite film as the emissive layer. The perovskite film consists of separated mixed halide perovskite phases with blue and red emissions, which are beneficial for suppressing halide anion exchange and preventing charge transfer. As a result, the white PeLED shows balanced white light emission with Commission Internationale de L'Eclairage coordinates of (0.33, 0.33). In addition, we find that the achievement of white light emission from mixed halide perovskites strongly depends on effective modulation of the halide salt precursors, especially lead bromide and benzamidine hydrochloride in our case. Our work provides very useful guidelines for realizing single-emissive-layer all-perovskite white PeLEDs based on mixed halide perovskites, which will spur the development of high-performance white PeLEDs.

## Abstract 2

We demonstrated a single-emissive-layer all-perovskite white light-emitting diode based on a mixed halide perovskite film.

## Graphical abstract

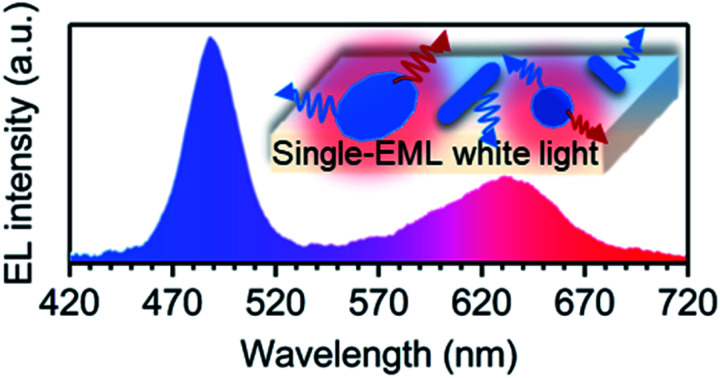

## Introduction

Metal halide perovskites are attracting considerable attention in the field of solution-processed light-emitting diodes (LEDs), due to their outstanding optical and electrical properties, such as high photoluminescence quantum efficiency (PLQE), tunable bandgap, and high color purity.^1–4^ These features have driven considerable research efforts to achieve high-performance perovskite LEDs (PeLEDs).^5–8^ At present, green, red, and near-infrared PeLEDs have achieved high external quantum efficiencies (EQEs) of over 20%,^9–12^ and a decent EQE of 12.3% is also realized for blue PeLEDs.^13^

Yet the development of white PeLEDs lags far behind their monochromatic counterparts. There are rare reports about white PeLEDs, especially all-perovskite ones (Table S1†), although efficient hybrid white LEDs with perovskite emitters coated on commercial LED chips have been achieved.^14^ Some low-dimensional lead-based perovskites featuring broad white PL from self-trapped excitons (STEs) have been reported,^15–17^ whereas no white electroluminescence (EL) has been demonstrated based on these materials. Similarly, strong white PL from STEs was observed in alloyed double perovskites, yet with low efficiency of white EL emission.^18^

Considering the high efficiencies achieved in the Br/Cl-based blue,^19^ Br-based green,^9^ and Br/I-based red PeLEDs,^10^ it would be fruitful if these highly emissive species could be used to fabricate all-perovskite white PeLEDs. Generally, white PeLEDs require a device architecture with either a single white emissive layer (EML)^20^ or multiple EMLs of three primary colors (or two complementary colors).^21^ Multiple-EML structures with three primary colors require orthogonal solvents to process the perovskite EMLs; unfortunately, perovskite precursors are only soluble in a few polar solvents.^22^ An alternative strategy is to fabricate dual-color white PeLEDs by combining polycrystalline perovskite films (processed from polar solvents) and colloidal perovskite nanocrystals (processed from non-polar solvents). Recently, a white PeLED with such a multiple-EML structure has been reported, which consists of a red two-dimensional (2D) perovskite EML and a cyan perovskite quantum dot EML separated by an organic interlayer.^23^

Despite this success, single-EML structures are more desirable for white PeLEDs due to much-simplified device structures and easy processing conditions compared with the multiple-EML counterparts.^24^ However, this approach has been very challenging due to two issues: (a) a single mixed halide perovskite film with blended Br/Cl-based blue perovskites, Br-based green perovskites and Br/I-based red perovskites tend to show very fast anion exchange;^25^ (b) there can be fast charge transfer from high- to low-energy emissive species in a blend film.^7,26^ These two issues will eventually result in monochromatic emission in the mixed halide perovskite film. Therefore, there is no report on single-EML all-perovskite white PeLEDs based on mixed halide perovskites so far.

Here, we demonstrate a first attempt to develop a single-EML all-perovskite white PeLED that employs a mixed halide perovskite film as the EML. The mixed halide perovskite film features segregated CsPb(Br_1−*x*_Cl_*x*_)_3_ and CsPb(Br_1−*y*_I_*y*_)_3_ grains; this unique film shows suppressed anion exchange and prevented charge transfer, leading to balanced dual-color white EL with Commission Internationale de L'Eclairage (CIE) coordinates of (0.33, 0.33) and an external quantum efficiency (EQE) of 0.008%. In addition, we find that the presence of both benzamidine hydrochloride (BHCl) and PbBr_2_ in the precursor solution, which can form low-dimensional intermediate phases, is responsible for the formation of the segregated CsPb(Br_1−*x*_Cl_*x*_)_3_ and CsPb(Br_1−*y*_I_*y*_)_3_ grains. Our work highlights the importance of effective modulation of mixed halide perovskite precursors for achieving dual-color white light emission and provides useful guidelines for the future development of high-performance single-EML white PeLEDs.

## Results and discussion

**Fig. 1:**
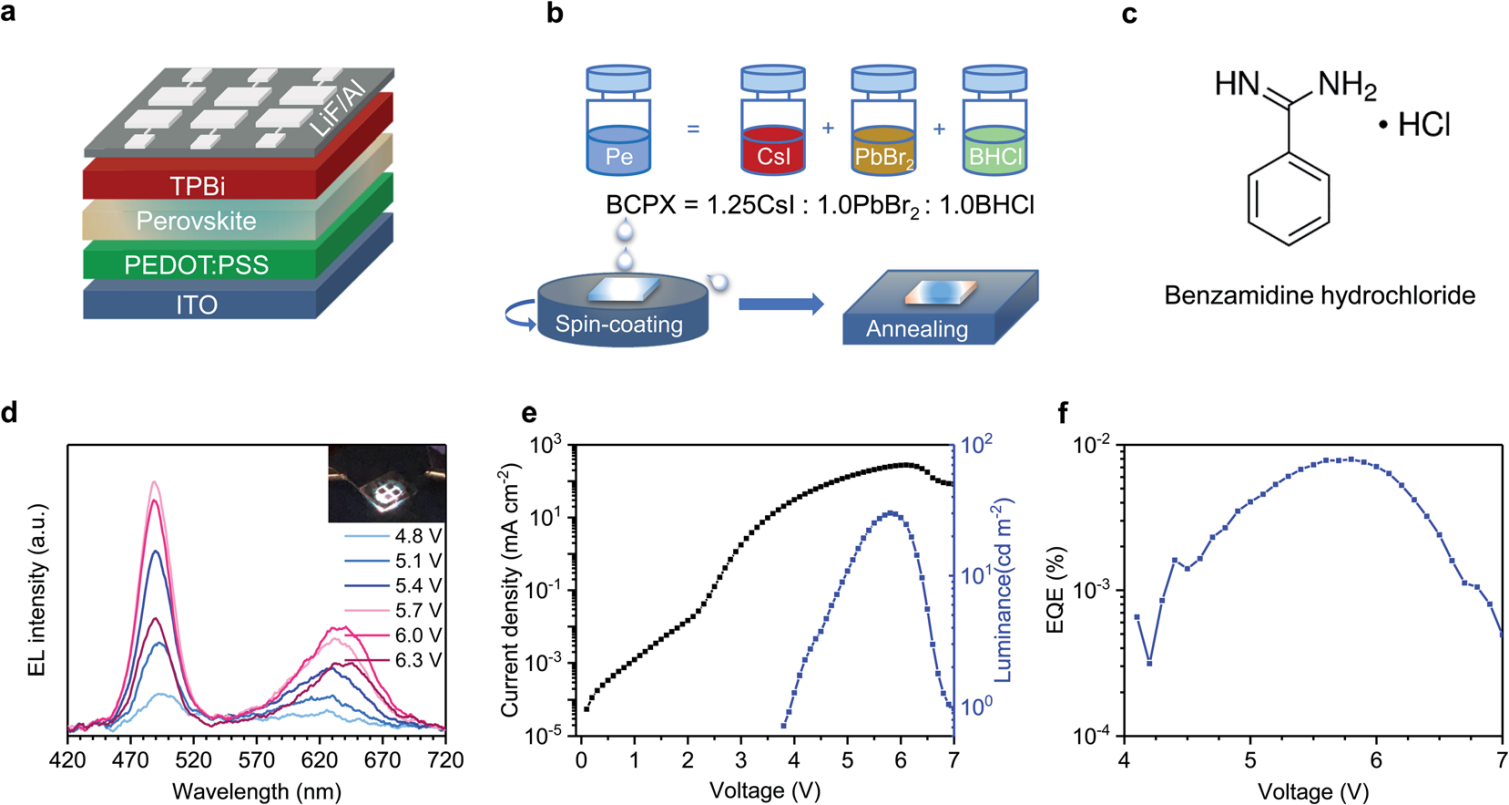
(a) Device architecture of the single-EML white PeLED. (b) Schematic diagram for the preparation of precursor solution and BCPX film. (c) Chemical structure of benzamidine hydrochloride (BHCl). (d) EL spectra at different driving voltages (inset: photograph of a working device), (e) current density–voltage–luminance, and (f) EQE–voltage curves of the single-EML white PeLED.

Fig. 1a shows the device architecture of our white PeLEDs: ITO/PEDOT:PSS (40 nm)/perovskite (30 nm)/TPBi (35 nm)/LiF (1 nm)/Al (100 nm), where ITO, PEDOT:PSS and TPBi are indium tin oxide, poly(3,4-ethylenedioxythiophene) polystyrene sulfonate, and 2,2′,2′′-(1,3,5-benzinetriyl)-tris(1-phenyl-1-*H*-benzimidazole), respectively. The schematic diagram for the preparation of the perovskite (denoted as BCPX) precursor solution and film is shown in Fig. 1b. CsI, PbBr_2_, and an ammonium chloride salt benzamidine hydrochloride (BHCl, chemical structure presented in Fig. 1c) are dissolved in dimethyl sulfoxide (DMSO), respectively, and then mixed with a molar ratio of 1.25 : 1.0 : 1.0 to form the precursor solution. The BCPX film is then deposited by one-step spin-coating from the precursor solution, followed by thermal annealing at 80 °C for 40 min (see Experimental section for details).

The resulting BCPX film exhibits dual-color white light emission during device operation. Fig. 1d shows the EL spectra of the BCPX-based device at various driving voltages. In contrast to the conventional mixed halide perovskites which show monochromatic emission,^25^ this device exhibits broad emission with two peaks at 488 nm and 630 nm, respectively. The insert of Fig. 1d is a photograph of this device in operation, demonstrating the achievement of a single-EML white PeLED. This is further verified by the CIE coordinates, which are (0.33, 0.33) at 5.8 V (Table S2†). Fig. S1† shows evolution of the EL spectrum of the device operated at a constant current density of 100 mA cm^−2^. The low-energy emission peak in these EL spectra shows continuous redshift, which could be attributed to halide segregation as demonstrated in previous studies.^26,27^ In addition, the EL intensities of the blue and red components change over time, which is a common observation for single-EML white LEDs.^28^ The current density–voltage–luminance characteristics and EQE–voltage curve of this device are shown in Fig. 1e and f, respectively, showing a maximum luminance of 30 cd m^−2^ and a peak EQE of 0.008% at 5.8 V (253 mA cm^−2^).

**Fig. 2:**
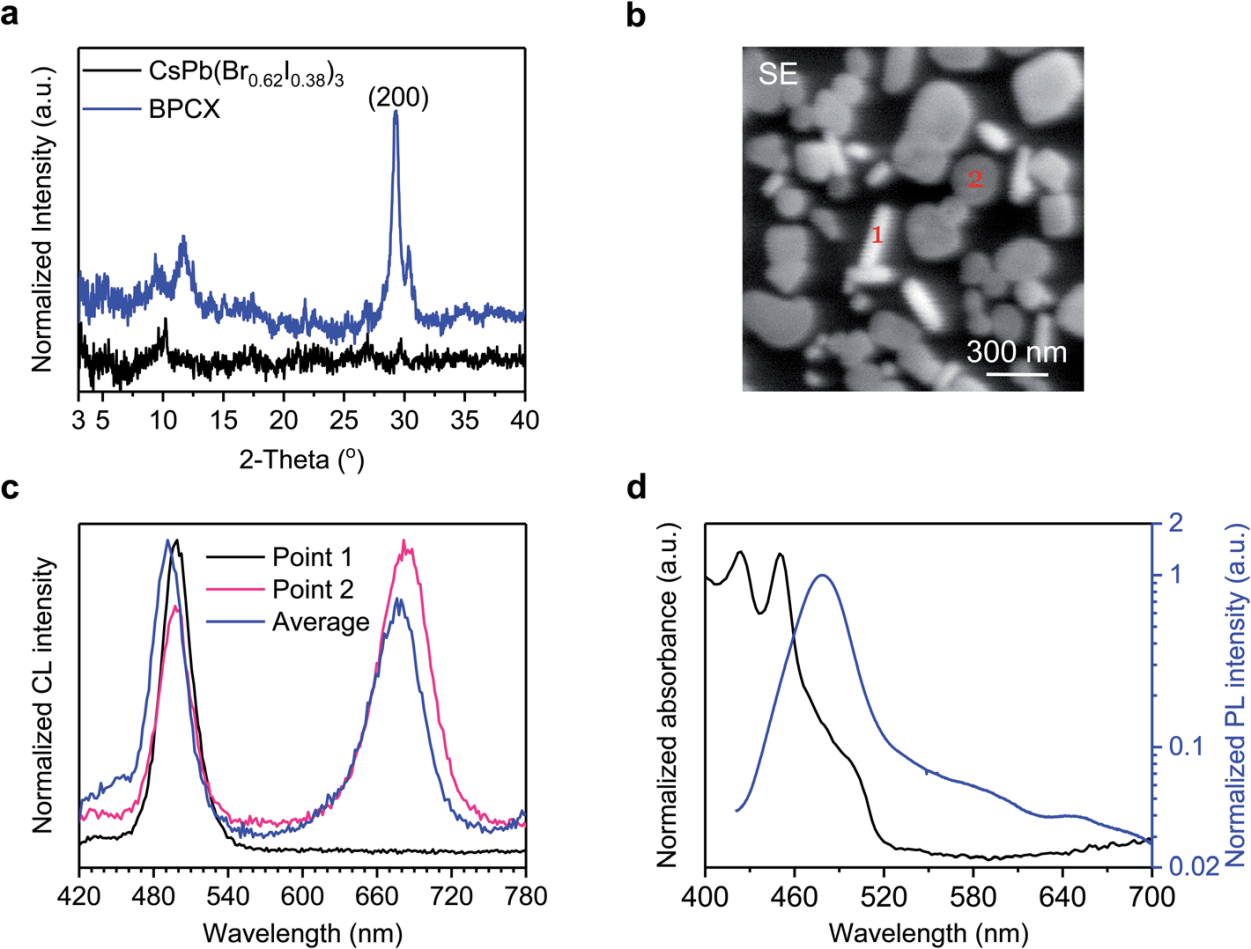
(a) XRD patterns of CsPb(Br_0.62_I_0.38_)_3_ and BCPX perovskite films. (b) Secondary electron (SE) image, and (c) cathodoluminescence (CL) spectra of the BCPX film acquired at 5.0 keV. (d) UV-vis absorption and PL spectra of the BCPX perovskite film.

The achievement of white EL from such a mixed halide perovskite film inspires us to investigate how the white light is generated. We first conduct X-ray diffraction (XRD) measurements to investigate the crystal structures of the BCPX film with the CsPb(Br_0.62_I_0.38_)_3_ film as a control (Fig. 2a). The CsPb(Br_0.62_I_0.38_)_3_ film is prepared following the synthetic procedures in Fig. 1b yet without using BHCl. No obvious diffraction peak from the perovskite phase is detected for the CsPb(Br_0.62_I_0.38_)_3_ film, which may be attributed to the low annealing temperature of 80 °C, since Cs-based I-rich perovskites such as CsPbBr_2_I usually require a high annealing temperature (∼300 °C).^29^ In the XRD pattern of the BCPX film, two characteristic diffraction peaks for perovskite phases at 29.3° and 30.3° are observed. It has been reported that CsPbI_3_, CsPbBr_3_, and CsPbCl_3_ have the (200) crystal planes at 28.6°, 30.1°, and 30.9°,^30^ respectively. The peak splitting for the (200) plane, that is, 29.3° and 30.3°, possibly indicates the coexistence of CsPb(Br_1−*y*_I_*y*_)_3_ (2*θ* = 29.3°)^31^ and CsPb(Br_1−*x*_Cl_*x*_)_3_ (2*θ* = 30.3°)^32^ perovskite phases in the BCPX film. Note that CsPb(Br_1−*y*_I_*y*_)_3_ and CsPb(Br_1−*x*_Cl_*x*_)_3_ perovskites are red and blue emissive species, respectively.^4^ The coexistence of the CsPb(Br_1−*y*_I_*y*_)_3_ and CsPb(Br_1−*x*_Cl_*x*_)_3_ perovskite phases in the BCPX film agrees well with the observation of its dual-color white EL, where the red emission is at 630 nm and the blue emission is at 488 nm (Fig. 1d). The formation of CsPb(Br_1−*y*_I_*y*_)_3_ perovskites at low annealing temperature of 80 °C can be attributed to the presence of BHCl.^33,34^

To further understand the CsPb(Br_1−*x*_Cl_*x*_)_3_ and CsPb(Br_1−*y*_I_*y*_)_3_ perovskite phases in the BCPX film, we carry out morphological investigations and cathodoluminescence (CL) measurements. Fig. 2b shows the secondary electron (SE) image of the BCPX film, which consists of obviously separated perovskite grains with different shapes, rod-like grains (such as Point 1) and plate-like grains (such as Point 2). Interestingly, these two types of grains deliver different CL (Fig. 2c). The rod-like grain (Point 1) only shows high-energy emission with a peak wavelength of 498 nm, suggesting that CsPb(Br_1−*x*_Cl_*x*_)_3_ perovskites dominate in such grains. The plate-like grain (Point 2) exhibits dual emission peaks, both at 498 and 684 nm, indicating the coexistence of CsPb(Br_1−*x*_Cl_*x*_)_3_ and CsPb(Br_1−*y*_I_*y*_)_3_ domains in these grains. These observations are in good agreement with the XRD results, confirming that there are segregated CsPb(Br_1−*x*_Cl_*x*_)_3_ and CsPb(Br_1−*y*_I_*y*_)_3_ crystals in the BCPX film. In addition, the average CL spectrum from the whole observation area also presents dual emission peaks at 491 and 677 nm, which are close to those of Point 1 and 2. The low-energy emission peak from CsPb(Br_1−*y*_I_*y*_)_3_ domains in CL (Fig. 2c) redshifts compared with those in EL (Fig. 1d), which might result from the much higher carrier density in the CL process.^35^ In addition, the BCPX film demonstrates good beam-radiation tolerance,^36^ which only shows minor damage after beam exposure for 5 min (Fig. S2a†), together with a slight spectral redshift (4 nm) of the low-energy emission peak (Fig. S2b†). These results confirm that the BCPX film allows for segregated CsPb(Br_1−*x*_Cl_*x*_)_3_ and CsPb(Br_1−*y*_I_*y*_)_3_ perovskite crystals, between which halide exchange is suppressed.

In addition, we find that charge transfer from the blue-emitting CsPb(Br_1−*x*_Cl_*x*_)_3_ to the red-emitting CsPb(Br_1−*y*_I_*y*_)_3_ perovskite crystals is prevented. The ultraviolet-visible (UV-vis) absorption and PL spectra of the BCPX film are shown in Fig. 2d. The absorption mainly takes place in the range of 400–520 nm, attributed to the CsPb(Br_1−*x*_Cl_*x*_)_3_ perovskites.^4^ The lack of significant absorption from CsPb(Br_1−*y*_I_*y*_)_3_ indicates that the amount of CsPb(Br_1−*y*_I_*y*_)_3_ is small compared with CsPb(Br_1−*x*_Cl_*x*_)_3_. The PL spectrum is dominated by high-energy emission with the peak wavelength at 479 nm although a weak red-emission peak at around 650 nm can be observed, suggesting that the charge transfer from CsPb(Br_1−*x*_Cl_*x*_)_3_ to CsPb(Br_1−*y*_I_*y*_)_3_ perovskites is not efficient during photo-excitation. We notice that the red-emissive PL, CL, and EL locate at slightly different positions, possibly due to emission shift induced by different carrier densities of mixed halide perovskites for these three different measurements. The lack of efficient charge transfer is further confirmed by ultrafast transient absorption (TA) measurement (Fig. S3†). The TA spectra of the BCPX film mainly show two ground-state bleach peaks (GSB 1 and GSB 2) at 454 nm and 497 nm. Note that no significant charge transfer from the CsPb(Br_1−*x*_Cl_*x*_)_3_ to CsPb(Br_1−*y*_I_*y*_)_3_ domains is observed, which is possibly due to the discontinuous film morphology of the BCPX film, where the CsPb(Br_1−*x*_Cl_*x*_)_3_ based rod-like grains are isolated to the plate-like grains. Such suppressed charge transfer ensures the presence of sufficient blue emission to realize white light emission.

**Fig. 3:**
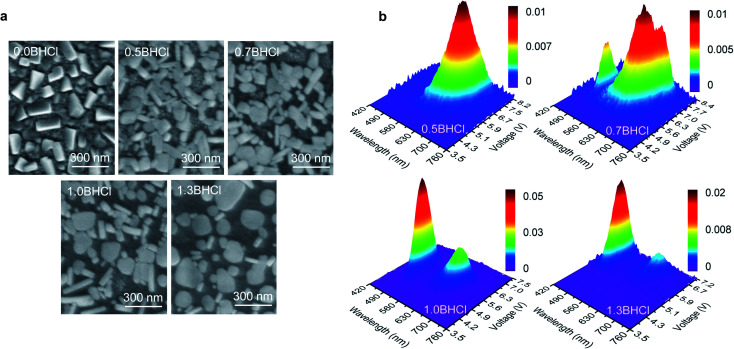
(a) SEM images for *m*BHCl (*m* = 0.0, 0.5, 0.7, 1.0, and 1.3) films. (b) 3D surface images of EL spectra for devices based on *m*BHCl (*m* = 0.5, 0.7, 1.0, and 1.3) films.

To further understand the formation mechanism of such morphology that leads to the white emission, we fabricate more perovskite films with various amounts of BHCl, which are denoted by *m*BHCl (*m* = 0.0, 0.5, 0.7, 1.0, and 1.3, which is the molar ratio of BHCl used; the 0.0BHCl and 1.0BHCl films are actually the above-mentioned CsPb(Br_0.62_I_0.38_)_3_ and BCPX films, respectively). As shown in Fig. 3a, the scanning electron microscopic (SEM) images of these films show obvious morphological variations as the amount of BHCl increases. The 0.0BHCl film shows discontinuous morphology yet with perovskite grains in the same shape. With BHCl addition, the grain shape changes and rod-like grains appear. Obviously, increasing the fraction of BHCl facilitates the formation of further separated rod- and plate-like grains. As discussed above, rod- and plate-like grains show different perovskite domains and consequently different emission behaviors. Such variations in morphology are expected to change the EL behaviors of these films.

3D surface images in Fig. 3b present EL spectral characteristics of devices based on the *m*BHCl (*m* = 0.5, 0.7, 1.0, and 1.3) films. The 0.0BHCl film based device shows no EL, which may be due to the above-mentioned fact that the low annealing temperature of 80 °C is unfavorable for the formation of CsPb(Br_1−*y*_I_*y*_)_3_ perovskite phases. Red EL spectra are observed in the 0.5BHCl film based device, indicating that the BHCl incorporation can promote the formation of CsPb(Br_1−*y*_I_*y*_)_3_ perovskites. By increasing the fraction of BHCl (*m* = 0.7), blue emission from CsPb(Br_1−*x*_Cl_*x*_)_3_ perovskites appears. Further increasing the content of BHCl (*m* = 1.0 and *m* = 1.3) induces dominant blue EL. The normalized EL spectra of these devices are presented in Fig. S4† for a better comparison. Apparently, the 1.0BHCl film, of which the I : Br : Cl molar ratio is 1.25 : 2.0 : 1.0, has led to a white PeLED with CIE coordinates close to those (0.3128, 0.3290) of the CIE standard illuminant D65 (Table S2†).

**Fig. 4:**
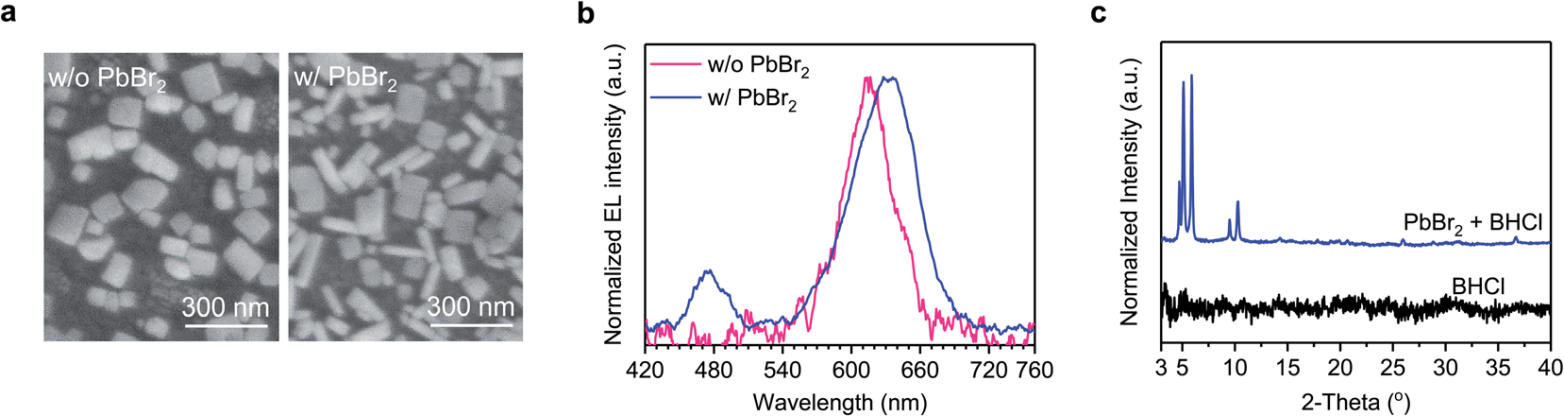
(a) SEM images of perovskite films without and with PbBr_2_. (b) Normalized EL spectra of devices based on perovskite films without and with PbBr_2_. (c) XRD patterns of pure BHCl and PbBr_2_ + BHCl mixed films.

Moreover, we find that the coexistence of PbBr_2_ and BHCl, providing Br and Cl sources, respectively, is key to ensure the formation of blue-emitting CsPb(Br_1−*x*_Cl_*x*_)_3_ rod domains for achieving the dual-color white light emission. We design an experiment without PbBr_2_, where Br is provided by CsBr, that is PbI_2_ : CsBr : BHCl = 1.0 : 1.25 : 1.0. As shown in Fig. 4a, only one type of grains is observed and the device exhibits monochromatic EL at 617 nm (without blue emission) (Fig. 4b). In contrast, both the emission and morphology show significant changes when 10 mol% PbI_2_ is replaced by PbBr_2_, that is, PbI_2_ : PbBr_2_ : CsBr : BHCl = 0.9 : 0.1 : 1.25 : 1.0. With 10 mol% PbBr_2_, both rod- and plate-like grains emerge in the film (Fig. 4a), and the device exhibits dual-color EL at 475 and 633 nm (Fig. 4b). This comparison confirms that the presence of PbBr_2_ can promote the formation of CsPb(Br_1−*x*_Cl_*x*_)_3_ domains with the participation of BHCl. Note that before and after PbBr_2_ replacement, the fraction of Br increases with changing the molar ratio of I : Br : Cl from 2.0 : 1.25 : 1.0 to 1.8 : 1.45 : 1.0. However, compared with the film without PbBr_2_, the film with PbBr_2_ shows a redshift in the low-energy emission instead of a blue-shift, implying a higher *y* value in the CsPb(Br_1−*y*_I_*y*_)_3_ species even though with more Br loading. This can be explained by the formation of the CsPb(Br_1−*x*_Cl_*x*_)_3_ species that consume parts of Br, resulting in less fraction of Br in the CsPb(Br_1−*y*_I_*y*_)_3_ species. These observations suggest that, in the presence of BHCl, the use of PbBr_2_ plays a significant role in the formation of CsPb(Br_1−*x*_Cl_*x*_)_3_ species.

Given that both BHCl and PbBr_2_ play key roles in the formation of the blue-emitting rod-shaped CsPb(Br_1−*x*_Cl_*x*_)_3_, we carry out XRD measurements on PbBr_2_ + BHCl film to further understand their impacts. As shown in Fig. 4c, XRD diffraction peaks at low angles (<7.5°) are observed in this film, suggesting the formation of low-dimensional phases. Given that these low-dimensional phases disappear in the BCPX (1.0BHCl) film (Fig. 2a), they may act as intermediate phases that control the growth process of the perovskite crystals. In contrast, the XRD pattern of CsBr + BHCl film only shows the diffraction peak from CsBr (Fig. S5†), suggesting that there is no new phase formed between CsBr and BHCl. We consider that the intermediate phases between BHCl and PbBr_2_ with the co-existence of Br and Cl are critical to the formation of blue-emitting CsPb(Br_1−*x*_Cl_*x*_)_3_.

In addition, our method is reproducible with well-selected additives. We further demonstrate the significant correlation between the unique morphology and dual-color emission by replacing BHCl of the BCPX film (Fig. 1b) with formamidine hydrochloride (FACl), acetamidine hydrochloride (AACl), and 4-fluoro-benzamidine hydrochloride (FBHCl, Fig. S6a†), respectively; the resulting films are denoted as FCPX, ACPX, and FBCPX, respectively. The corresponding SEM images of these films (Fig. S6b–d†) reveal that FBHCl replacement induces similar morphology to the BCPX film, in which both rod-like and plate-like grains appear (Fig. S6d†). Accordingly, the dual-color emission is only observed in the FBCPX-based device (Fig. S7†), suggesting that the unique morphology with dual shapes of grains is of significant importance for the achievement of dual-color white emission.

## Conclusions

In summary, we have demonstrated a single-EML all-perovskite white PeLED with a mixed halide perovskite film as the EML. The perovskite film encompasses CsPb(Br_1−*x*_Cl_*x*_)_3_ and CsPb(Br_1−*y*_I_*y*_)_3_ grains. Such morphology not only suppresses halide anion exchange between CsPb(Br_1−*x*_Cl_*x*_)_3_ and CsPb(Br_1−*y*_I_*y*_)_3_ domains but also prevents charge transfer from the blue-emitting CsPb(Br_1−*x*_Cl_*x*_)_3_ to the red-emitting CsPb(Br_1−*y*_I_*y*_)_3_, allowing for dual-color white EL with CIE coordinates of (0.33, 0.33) and an EQE of 0.008%. The formation of CsPb(Br_1−*x*_Cl_*x*_)_3_ grains is found associated with the presence of both BHCl and PbBr_2_ in the precursor solution, indicating the importance of forming the intermediates with both Br and Cl. Although the device parameters still have large space to improve, our findings represent a new direction in achieving single-EML white PeLEDs using mixed halide perovskites, which will spur further development of high-performance single-EML white PeLEDs.

## Conflicts of interest

We declare no conflict of interest.

## Supplementary Material

SC-011-D0SC04508J-s001
