# Supplementary material for: Single-emissive-layer all-perovskite white light-emitting diodes employing segregated mixed halide perovskite crystals
Source: Chem Sci. 2020 Sep 25;11(41):11338–43. doi: 10.1039/d0sc04508j (PMC8162942; doi:10.1039/d0sc04508j)
Supplement: SC-011-D0SC04508J-s001 [file SC-011-D0SC04508J-s001.pdf]

Electronic Supplementary Material (ESI) for Chemical Science.
This journal is © The Royal Society of Chemistry 2020

Supporting Information

Single-Emissive-Layer All-Perovskite White Light-Emitting Diodes Employing Segregated Mixed Halide Perovskite Crystals

Hongling Yu, Heyong Wang, Galia Pozina, Chunyang Yin, Xiao-Ke Liu and Feng Gao**

Department of Physics, Chemistry and Biology (IFM), Linköping University, Linköping 58183, Sweden
E-mail: xiaoke.liu@liu.se; feng.gao@liu.se

Experimental Section

***Materials*:** CsI, CsBr, PbI_2, formamidine hydrochloride (FACl), acetamidine hydrochloride (AACl), and 4-fluoro-benzamidine hydrochloride (FBHCl), and benzamidine hydrochloride (BHCl) were purchased from Sigma-Aldrich. $PbBr_2$ (99.998%) and LiF were purchased from Alfa Aesar. PEDOT:PSS (AL4083) was purchased from Ossila. TPBi was purchased from Lumtec.

***Precursor solution preparation and film deposition*:** CsI, CsBr, PbI_2, $PbBr_2$ solutions of 0.2 M and BHCl solution of 1.0 M were dissolved in DMSO before mixing up for desired precursor solutions. Two groups of solutions with a final molar concentration of 0.075 M (Pb^{2+}) were prepared: (1) mixtures of 1.25CsI + 1.0$PbBr_2$ + *m*BHCl, donated as *m*BHCl (m = 0.0, 0.5, 0.7, 1.0, and 1.3); (2) mixtures of 1.0PbI_2 + 0$PbBr_2$ + 1.25CsBr + 1.0BHCl and 0.9PbI_2 + 0.1$PbBr_2$ + 1.25CsBr + 1.0BHCl. After mixed up, all the precursor solutions were kept overnight at room temperature before deposited into films. To form films, the precursor solutions were spin-coated in a N_2-filled glovebox at 3000 rpm for 60 s and then annealed at 80 ^{o}C for 40 min. The FCPX, ACPX, and FBCPX films were prepared as the BCPX film (1.0BHCl), where BHCl is completely replaced by FACl, AACl, and FBHCl, respectively.

***Film Characterization*:**

Ultraviolet-visible (UV-Vis) absorbance spectra were measured with a PerkinElmer model Lambda 900. Steady-state PL spectra (λ_{ex} = 405 nm) were recorded by an Andor spectrometer (Shamrock sr-303i-B, coupled to a Newton EMCCD detector). XRD patterns were acquired by using a Panalytical Empyrean system with Cu Kα source (λ = 1.54 Å).

Scanning electron microscopy (SEM, Philips XL30 FEG SEM) was operated at 3 keV to characterize the morphology of the samples. CL measurements were performed by a MonoCL4 system integrated with SEM. The acceleration voltage of the electron beam was kept at 5 keV,

which allows an acquisition of the CL signal with a spatial resolution of ~100 nm. A fast CCD detection system have been used for spectral detection.

Transient absorption (TA) measurements were conducted by a femtosecond TA (Mai Tai, Spectra Physics) in air atmosphere. The pump laser (800 nm, 80 fs pulse length, 1 kHz repetition rate) was generated by a regenerative amplifier (Spitfire XP Pro). The second harmonic generation (400 nm) from a BBO crystal was used as pump. For the probe, the super-continuum generation from a thin CaF_2 plate was used.

***Device fabrication and characterization*:** ITO-coated glasses were cleaned for 15 min in the ultrasonic bath and then dried by the nitrogen flow. After a further clean up by a ultraviolet-ozone for 10 min, filtered PEDOT:PSS solution was deposited onto the ITO substrates at 4000 rpm for 30 s, followed by thermal annealing at 140 ^{o}C for 15 min in the air. Then the substrates were transferred into the N_2-filled glovebox for perovskite films deposition. The preparation of perovskite layers followed the same procedures of film deposition described above. The TPBi (35 nm), LiF (1 nm), and Al (100 nm) layers were deposited at the rates of 1.0 Å/s, 0.2 Å/s, and 3.0 Å/s, respectively, at a vacuum of 2×10^{-6} Torr. The current density and the driving voltage were collected by a Keithley 2400 source meter, swept from zero bias with a step of 0.1 V. Each step took 0.8 s for stabilization measurements. The light emission was collected by an integrating sphere together with a QE Pro spectrometer (Ocean Optics). The active device area is 0.0725 cm^2. All the device characterizations were carried out at room temperature in a nitrogen-filled glovebox.

Table S1. Summary of the state-of-the-art white LEDs with EMLs containing perovskites.

Approach	Emissive layer(s)	CIE (x, y)	Luminance (cd m^{-2})	EQE (%)	Ref.
**Multiple-EML**	EML 1: $PA_2CsPb_2I_7$<br>EML 2: $CsPb(Br/Cl)_3$	(0.32, 0.32)	-	0.22	1
**Single-EML**	$CsPbBr_xCl_{3-x}$ mixed with MEH:PPV	(0.33, 0.34)	80	-	2
**Single-EML**	$Cs_2Ag_{0.60}Na_{0.40}InCl_6$	-	~ 50	-	3
**Single-EML**	$CsPb(Br_{1-x}Cl_x)_3$ and $CsPb(Br_{1-y}I_y)_3$ blend	(0.33, 0.33)	30	0.008	This work

MEH:PPV: poly[2-methoxy-5-(2-ethylhexyloxy)-1,4-phenylenevinylene]

Table S2. Peak wavelengths and CIE coordinates of our white PeLEDs at different driving voltages.

Driving voltage (V)	Peak wavelength (nm)	CIE coordinate (*x, y*)
**4.8**	493 + 626	(0.35, 0.45)
**5.1**	493 + 630	(0.34, 0.38)
**5.4**	490 + 630	(0.32, 0.37)
**5.5**	489 + 631	(0.32, 0.34)
**5.6**	488 + 632	(0.32, 0.33)
**5.7**	488 + 632	(0.33, 0.33)
**5.8**	488 + 632	(0.33, 0.33)
**5.9**	488 + 631	(0.34, 0.32)
**6.0**	488 + 631	(0.35, 0.32)
**6.3**	490 + 646	(0.38, 0.32)

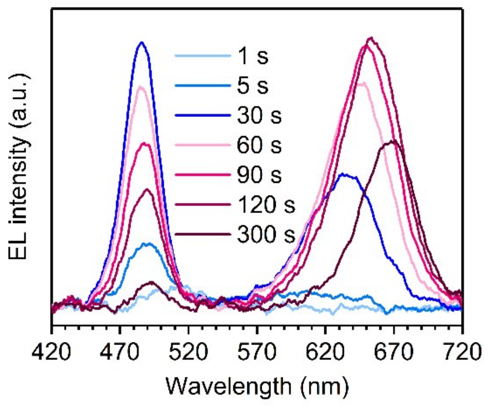

Fig. S1 Evolution of EL spectrum of the BCPX-based device; the device continuously operated at a constant current density of 100 mA cm^{-2}.

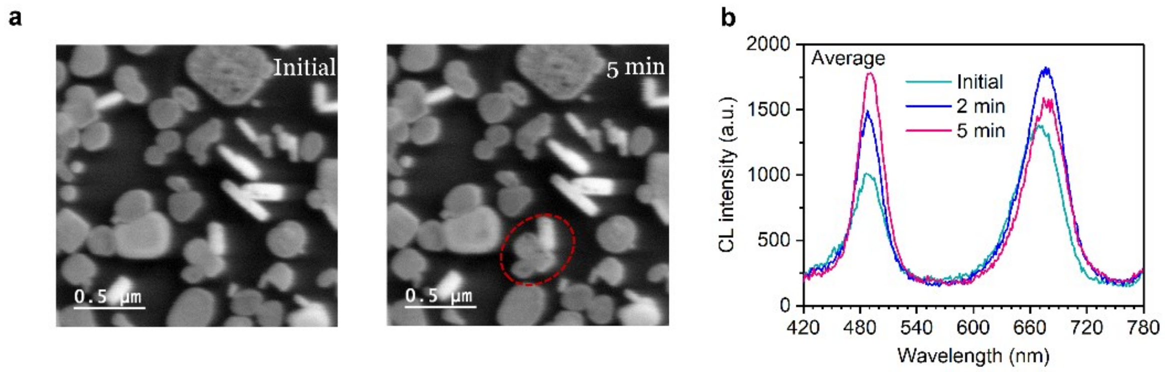

Fig. S2 (a) SE images and (b) CL spectra of the BCPX film acquired at 5.0 keV with prolonged beam exposure time. The grains in the red dash circle show minor damage.

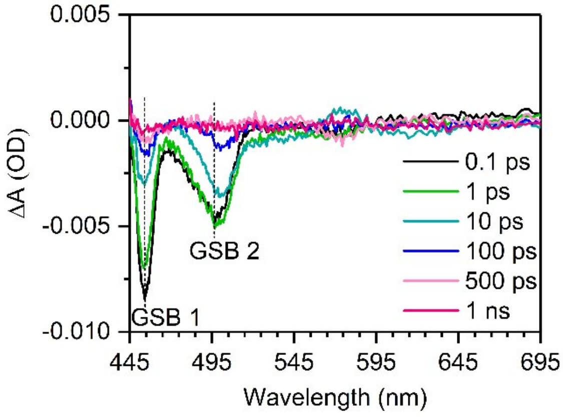

Fig. S3 TA spectra for the BCPX perovskite film.

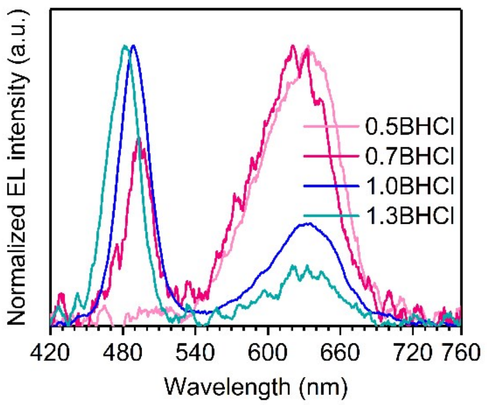

Fig. S4 Normalized EL spectra of devices based on *m*BHCl (*m* = 0.5, 0.7, 1.0, and 1.3) films.

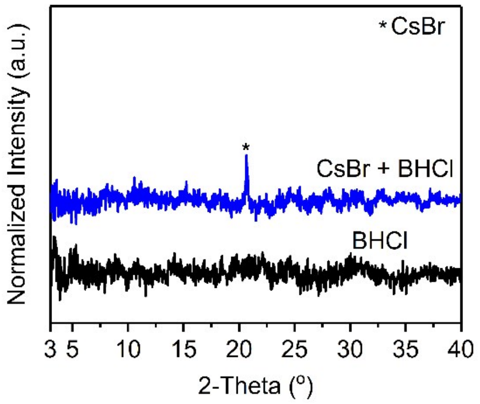

Fig. S5 XRD patterns of pure BHCl and CsBr + BHCl films. The peak marked by asterisk is from CsBr.[4]

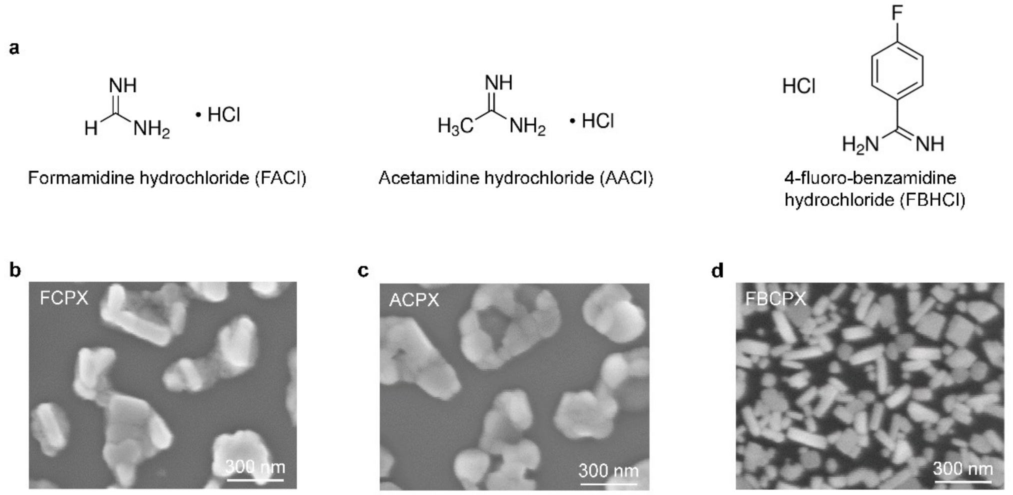

Fig. S6 (a) Chemical structures of formamidine hydrochloride, acetamidine hydrochloride, and 4-fluoro-benzamidine hydrochloride. (b) SEM images of the corresponding FCPX, ACPX, and FBCPX films.

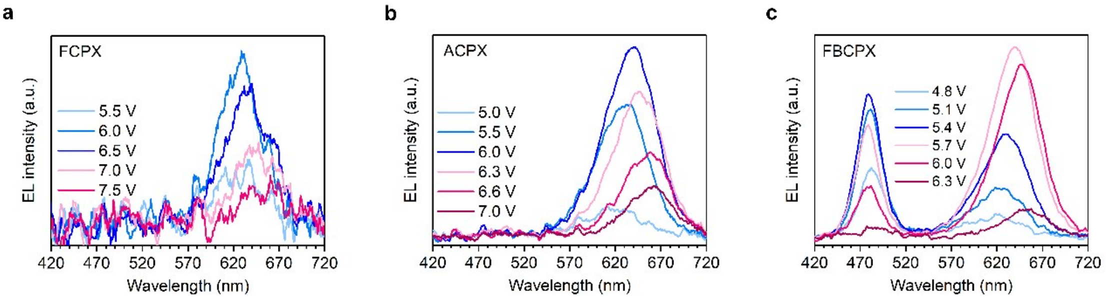

Fig. S7 EL spectra of devices based on (a) FCPX, (b) ACPX, and (c) FBHCl films at different driving voltages.

References

1. J. Mao, H. Lin, F. Ye, M. Qin, J. M. Burkhartsmeyer, H. Zhang, X. Lu, K. S. Wong and W. C. H. Choy, *ACS Nano* 2018, **12**, 10486-10492.
2. E.-P. Yao, Z. Yang, L. Meng, P. Sun, S. Dong, Y. Yang and Y. Yang, *Advanced Materials Adv. Mater.* 2017, **29**, 1606859.
3. J. Luo, X. Wang, S. Li, J. Liu, Y. Guo, G. Niu, L. Yao, Y. Fu, L. Gao, Q. Dong, C. Zhao, M. Leng, F. Ma, W. Liang, L. Wang, S. Jin, J. Han, L. Zhang, J. Etheridge, J. Wang, Y. Yan, E. H. Sargent and J. Tang, *Nature* 2018, **563**, 541-545.
4. Z. Hong, D. Tan, R. A. John, Y. K. E. Tay, Y. K. T. Ho, X. Zhao, T. C. Sum, N. Mathews, F. García and H. S. Soo, *iScience* 2019, **16**, 312-325.
